# Association between abdominal obesity and cognitive decline among Chinese middle-aged and older adults: a 10-year follow-up from CHARLS

**DOI:** 10.3389/fpubh.2025.1479355

**Published:** 2025-04-15

**Authors:** Qiong Wu, Xu Zhu, Dan Feng, Ziyan Zhang, Can Wen, Xinbin Xia

**Affiliations:** ^1^College of Humanities and Management, Hunan University of Chinese Medicine, Changsha, Hunan, China; ^2^College of Integrated Traditional Chinese and Western Medicine, Hunan University of Chinese Medicine, Changsha, Hunan, China; ^3^School of Public Health, Anhui Medical University, Hefei, Anhui, China; ^4^School of Public Administration, Central South University, Changsha, Hunan, China; ^5^First Affiliated Hospital of Gannan Medical University, Ganzhou, Jiangxi, China

**Keywords:** abdominal obesity, cognitive decline, CHARLS, HDL-C, mediation analysis

## Abstract

**Introduction:**

The relationship between abdominal obesity and cognitive decline has controversial results, and the mediating effect of high-density lipoprotein cholesterol (HDL-C) between them remains uncertain. This study aims to explore the association between abdominal obesity and cognitive decline in middle-aged and older adults, including dose-response relationship and age differences, as well as the mediating effect of HDL-C.

**Methods:**

Data were obtained from the China Health and Retirement Longitudinal Study (CHARLS), involving 3,807 participants aged 45 and above from 2010 to 2020. The TICS-10 was used to assess cognitive function, and the group-based trajectory model (GBTM) was used to explore the potential heterogeneity of cognitive changes. Abdominal obesity was measured by baseline waist circumference (WC) and a sequentially adjusted unordered multinomial logistic regression was used to investigate the association between abdominal obesity and cognitive decline in middle-aged and older adults. Restricted cubic spline (RCS) model was adopted to analyze the dose-response relationship between WC and risk of cognitive decline. HDL-C was used as a mediator to examine the potential causal chain between abdominal obesity and cognitive decline.

**Results:**

Among the 3,807 participants, a total of 1,631 individuals (42.84%) had abdominal obesity. The GBTM identified 3 cognitive function trajectories: rapid decline (11.0%), slow decline (41.1%) and stable groups (47.9%). After controlling for confounders, participants with abdominal obesity were less likely to experience rapid decline (*OR*: 0.67, 95%*CI*: 0.51–0.8) and slow decline (*OR*: 0.81, 95%*CI*: 0.69–0.95) of cognitive function, compared to those with normal WC. RCS analysis showed a decreased risk of cognitive decline with increasing WC. In the age subgroup analysis, the protective effect was significant only in the population aged 50 and above. HDL-C mediated 19.15% (*P* < 0.05) of the relationship between abdominal obesity and cognitive decline.

**Conclusion:**

Abdominal obesity had a significant protective effect on cognitive decline in Chinese middle-aged and older adults, with HDL-C playing a mediating role in the relationship between abdominal obesity and cognitive decline.

## 1 Introduction

Cognitive decline is a major global public health challenge, with severe cases leading to cognitive impairment and developing into dementia (1). As the seventh leading cause of death globally (2), dementia affects over 55 million individuals worldwide by 2023. China alone accounts for ~15.07 million dementia patients, ranking first globally. In China, dementia has emerged as one of the most expensive, lethal, and care-intensive diseases (3). Moreover, it is estimated that these figures will rise to 22.2 million and 29.89 million by 2030 and 2050 respectively (4). Dementia caused by cognitive impairment is irreversible, and demonstrate a trend toward affecting younger individuals, expanding the affected population from primarily 60 to 70 years old to middle-aged individuals aged 45 and above (5).

Obesity is a modifiable risk factor for cognitive disease such as dementia, including general obesity and abdominal obesity measured by Body Mass Index (BMI) (6). In the Chinese population, abdominal obesity is more prevalent than general obesity (5). Despite the availability of various metrics for measuring obesity, such as BMI, waist circumference (WC), body roundness index (BRI), visceral adiposity index (VAI), etc., the relationship between obesity, particularly abdominal obesity, and cognitive decline remains unclear (7, 8). A systematic review and meta-analysis study has shown that BMI and WC are consistently associated with cognitive decline, while the BRI and VAI did not demonstrate significant predictive value in most studies, negating the validity of the BRI and VAI in the research on the relationship between obesity and cognition (9). The study conducted by Mina et al. analyzed the data of more than 8,000 Asian individuals and pointed that a single index such as the visceral adiposity index could not fully and accurately reflect the complex relationship between obesity and cognitive function (10). Compared complex calculation progress and the requirement of many biochemical indicators like the indicators such as VAI and BRI, the WC, which can be directly measured based on the accumulation of abdominal fat as Chinese people age, appears to be extremely simple and intuitive (11).

Nevertheless, studies on the correlation between abdominal obesity and cognitive decline yields conflicting results. Uchida et al. identified a significant gender-adjusted increase in dementia risk associated with larger waist circumference (WC) and abdominal obesity (12), while Liang et al. proposed that larger WC is a protective factor for cognitive decline (13). Ren et al. found no link between WC and cognitive impairment (14). Is abdominal obesity a risk or a protective factor for cognitive decline? One possible explanation is that the relationship between abdominal obesity and cognitive decline is also influenced by a series of mediating factors that cause neuropathological changes (15), such as high-density lipoprotein cholesterol (HDL-C).

Previous studies indicate that people with abdominal obesity tend to have lower levels of HDL-C (16). The latest study in The Lancet shows that very high levels of HDL-C can detrimentally affect cognitive function (15). Nevertheless, further investigation is needed to conclusively determine whether HDL-C mediates the relationship between abdominal obesity and cognitive decline, and to what extent it influences this association.

This study investigated the association between abdominal obesity, measured by WC, and cognitive decline in middle-aged and older Chinese adults, using data from the China Health and Retirement Longitudinal Study (CHARLS). We employed restricted cubic spline (RCS) model to explore potential nonlinear relationship between WC and risk of cognitive decline, and use HDL-C as a mediating variable to provide a necessary supplement to the mechanism linking this relationship.

## 2 Materials and methods

### 2.1 Study design and participants

**Figure 1:**
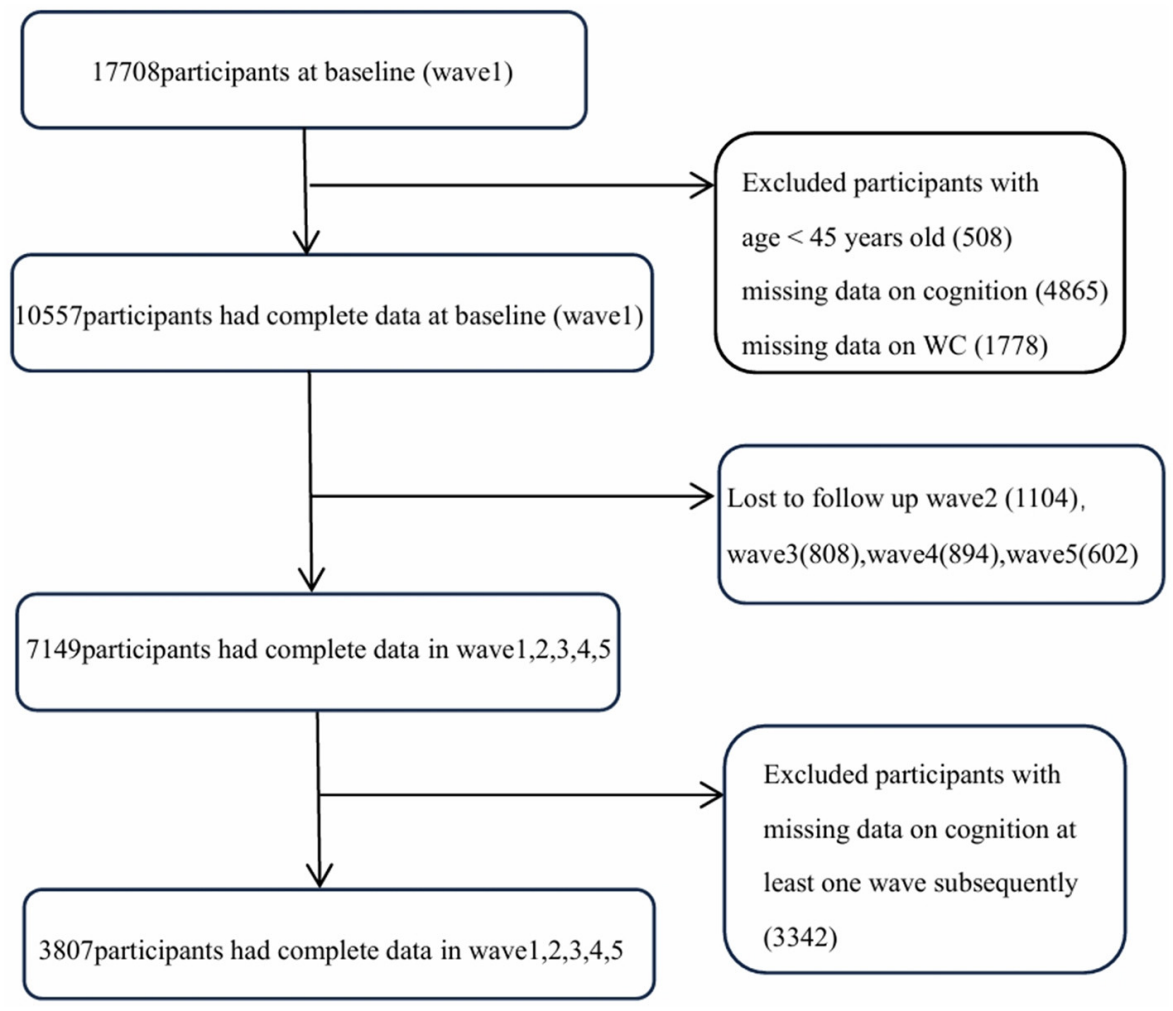
Flowchart for selecting study participants in this study.

The data for the study were derived from the China Health and Retirement Longitudinal Study (CHARLS), which is a national longitudinal social survey conducted by the Institute of Social Sciences at Peking University, focusing on individuals aged 45 and above in China. The baseline survey was conducted in 2011, involving 17,708 participants from 450 communities across 150 counties (districts) from 28 provinces in China. It involved 17,708 respondents from ~12,400 households, achieving an effective response rate of ~80%. Standardized questionnaire assessments were conducted through Computer Assisted Personal Interviewing (CAPI). The data collected encompassed various aspects, including personal demographic information, family structure, economic support, health status, and utilization of medical services (17). These respondents were followed up once every 2 years to repeat the survey, and five national waves of data are available to date (waves in 2011, 2013, 2015, 2018, and 2020)This study used data of spanning five waves (2011–2020) from CHARLS. A total of 17,708 participants were recruited at baseline, while 508 participants under the age of 45 were excuded, 6,643 individuals were excluded due to missing data on the cognitive assessment (4,865) and WC (1,778) at baseline. Outliers in WC were addressed using the 3σ rule. Additionally, 3,408 participants who did not complete follow-up and 3,342 participants missed at least one wave of cognitive assessment data subsequently were also excluded. Finally, a total of 3,807 participant were enrolled in the study (Figure 1).

### 2.2 Measures

#### 2.2.1 Cognitive function assessment

Cognitive function assessment encompass two dimensions broadly: episodic memory and mental state (1). According to the CHARLS (2011) User Manual, these two dimensions were from several measurements of the Telephone Interview of Cognitive Status (TICS) (18), such as recalling and delayed recall of 10 words; today's date, the day of the week, and the current season; the 100–7 calculation series; and drawing two repeated pentagons.

Episodic memory comprises two parts: immediate memory and delayed memory. The measurement of immediate memory requires the interviewer to read out a set of 10 Chinese nouns at a slow and steady pace, and then asks the respondent to immediately repeat these nouns. The measurement of delayed memory mainly refers to the interviewer reading out 10 Chinese nouns, and the respondent recalling and repeating as many of these words as possible after 4 min. The episodic memory score mainly refers to the average number of words successfully recalled and repeated immediately and after 4 min by the respondent, with the result score ranging from 0 to 10 (19).

Mental state includes other parts of the TICS scale borrowed by CHARLS (20). It mainly includes today's date, the day of the week, the current season; the 100-7 calculation series; and drawing two repeated pentagons. Inquiring the respondent about today's date, the day of the week, and the current season is used to assess the respondent's orientation ability, with a score ranging from 0 to 5. Measuring the respondent's ability to perform the 100–7 calculation series five times consecutively is used to assess attention, with a score ranging from 0 to 5. Assessing the respondent's ability to redraw the previously shown figures is used to evaluate the visual construction ability, with a score ranging from 0 to 1.

The overall cognitive score is the sum of the episodic memory and mental state tests. During the four waves spanning 2011 to 2018, all data collection was exclusively conducted through face-to-face interviews. During the wave 5, remote video interviews were systematically implemented. The cognitive function score ranges from 0 to 21, with lower scores indicating poorer cognitive ability (13).

#### 2.2.2 Abdominal obesity

Abdominal obesity is defined as WC ≥90 cm for men and WC ≥85 cm for women according to national standards (21) and CHARLS classification criteria (22). WC was objectively measured by trained investigators utilizing specialized equipment (23). The measurement was taken using a non-elastic, standardized tape measure positioned at the midpoint between the lowest rib and the ilium, ensuring that readings were obtained while the subject maintained calm breathing.

#### 2.2.3 HDL-C

The level of HDL-C in the blood were quantified utilizing an enzymatic colorimetric test. This procedure was carried out by trained personnels, who had undergone separate training sessions provided by the National CDC. These professionals were responsible for the collection, transportation, and testing of venous blood samples from the subjects, adhering to the “Blood Collection and Processing” protocol.

#### 2.2.4 Covariates

This study examined covariates including demographic factors, lifestyle habits, health status and biomarkers. Demographic factors included age, gender and education. Lifestyle habits included smoking and drinking. Health status included self-care abilities (assessed by activities of daily living and instrumental activities of daily living), depression, and presence of chronic diseases (self-reported and clinically measured hypertension and diabetes). Additionally, biomarkers included low-density lipoprotein (LDL-C) and triglycerides (TG).

### 2.3 Statistical analysis

Continuous variables were reported using means and standard deviations (*SDs*), while categorical variables were presented through frequencies and composition ratios. The characteristics of each variable were analyzed based on categories of abdominal obesity, as determined by WC. Univariate analysis was performed using the χ^2^test and *Mann-Whitney U* test.

Cognitive function trajectories were identified by Group-based trajectory modeling (GBTM), a semi-parametric grouping-based latent class growing model designed for longitudinal data analysis and heterogeneity exploration (24). The principle is to assume that there is heterogeneity, and there are several potential subgroups with different development trajectories in the population, and the purpose is to explore how many subgroups with different development trends are included. The process of identifying the optimal trajectory is an iterative process, considering various statistical measures, such as statistical indicators (the *p*-values of model parameters and the 95% confidence intervals), visual assessments of trajectory estimates, the smaller Bayesian information criterion (*BIC*), and the average posterior probability (*Avepp*), which should be above 0.7 indicated optimal fit, the allocation proportion of each group, which should be above 5% (25). We used the Stata Traj plug-in to display cognitive function trajectories from 2011 to 2020.

An unordered multinomial logistic regression, adjusted sequentially, was utilized to examine the correlation between abdominal obesity and cognitive function trajectories. Logistic regression analysis were conducted using three models: Model 1 was unadjusted, Model 2 was adjusted for age, sex, education, smoke and drink, Model 3 was further adjusted for ADL, IADL, depression, chronic disease, and biomarkers. WC was incorporated into the model as a numerical and subtype variable successively. The restricted cubic spline (RCS) served as an essential supplement to this association. RCS is a type of spline function utilized for flexibly modeling nonlinear relationships within regression models. Cubic splines (CS) employ cubic polynomials and achieve smooth functions by imposing the equality of the first and second derivatives at each knot. In this process, the values of the explanatory variable are transformed into new variables (a linear term, a quadratic term, a cubic term, and other truncated cubic polynomials), which serve as basis functions for estimating regression coefficients. Its function lies in effectively capturing the nonlinear relationships between explanatory variables and outcomes, avoiding overfitting risks through fitting curves in different intervals and optimizing knot settings to balance complexity and goodness of fit. Subsequently, it facilitates the exploration of causal relationships and the interpretation of results, providing crucial support for data analysis, prediction, and decision-making in regression models (26).

We utilized HDL-C as a mediating variable to explore its mediating effects on the relationship between abdominal obesity and cognitive function trajectories. Based on the criteria established by Baron and Kenny, we conducted mediation analysis using the mediation package to determine the total effect, direct effect, indirect effect values, and the proportion of the mediated effect. The bootstrap method was employed to assess the significance of the mediated effect, with random sampling performed 1,000 times (26).

Data cleaning, single factor analysis, trajectory fitting, and logistic regression analysis were all conducted using Stata18.0. RCS model construction and drawing, mediation analysis were performed using R4.4.0. Unless otherwise specified, the significance level throughout the text was two-sided α = 0.05.

## 3 Results

### 3.1 Baseline characteristics of the study participants

A total of 3,807 participant were enrolled in the study, and 1,631 people were classified as abdominal obesity (42.84%). The average age was (56.31 ± 7.55) years old, 2,117 were male (55.61%), most were in middle or high school (1,239, 32.56%), 1,624 had hypertension (42.75%), and 427 had diabetes (11.31%). 1,135 people were depressed (29.81%), the average HDL-C (50.23 ± 15.10) mg/dl, LDL-C (117.59 ± 34.22) mg/dl, and TG (135.96 ± 124.59) mg/dl. Baseline characteristics are shown in Table 1.

**Table 1:** Baseline characteristics of 3,807 participants by waist circumference category.

**Characteristic**	***N* (%)**	**Normal (2,176)**	**Obesity (1,631)**	***P*-value^a^**
Age, mean (SD^b^)	56.31 ± 7.55	56.42 ± 7.47	56.17 ± 7.66	0.218
**Gender**				
Male	2,117 (55.61)	1,403 (64.48)	714 (43.78)	<0.001
Female	1,690 (44.39)	773 (35.52)	917 (56.22)	
**Education** ^c^				
No formal level	837 (22.00)	474 (21.80)	363 (22.26)	0.825
Primary school	1,045 (27.46)	610 (28.06)	435 (26.67)	
Middle or high	1,239 (32.56)	702 (32.39)	537 (32.92)	
College and above	684 (17.98)	388 (17.85)	296 (18.15)	
**Smoke**				
Yes	1,260 (33.10)	875 (40.21)	385 (23.61)	<0.001
No	2,547 (66.90)	1,301 (59.79)	1,246 (76.39)	
**Drink**				
Yes	1,452 (38.14)	917 (42.14)	535 (32.80)	<0.001
No	2,355 (61.86)	1,259 (57.86)	1,096 (67.20)	
**ADL** ^b, c^				
Yes	405 (10.73)	211 (9.76)	194 (12.03)	0.026
No	3,369 (89.27)	1,950 (90.24)	1,419 (87.97)	
**IADL** ^b^				
Yes	463 (12.16)	258 (11.86)	205 (12.57)	0.506
No	3,344 (87.84)	1,918 (88.14)	1,426 (87.43)	
**Hypertension** ^c^				
Yes	1,624 (42.75)	705 (32.49)	919 (56.41)	<0.001
No	2,175 (57.25)	1,465 (67.51)	710 (43.59)	
**Diabetes** ^c^				
Yes	427 (11.31)	161 (7.45)	266 (16.47)	<0.001
No	3,348 (88.69)	1,999 (92.55)	1,349 (83.53)	
**Depression**				
Yes	1,135 (29.81)	677 (31.11)	4,580 (28.08)	0.043
No	2,672 (70.19)	1,499 (68.89)	1,173 (71.92)	
HDL-C^b, c^, mean (SD^b^), mg/dl	50.23 ± 15.10	53.76 ± 15.55	45.35 ± 35	<0.001
LDL-C^b, c^, mean (SD^b^), mg/dl	117.59 ± 34.22	114.63 ± 33.41	118.96 ± 35.15	<0.001
TG^c^, mg/dl	135.96 ± 124.59	115.83 ± 90.82	166.86 ± 151.15	<0.001

^*a*^P Value: based on χ ^2^ or Mann-Whitney U test where appropriate.

^*b*^SD, standard deviation. Adl, activities of daily living; Iadl, instrumental activities of daily living; HDL-C, high density lipoprotein cholesterol; LDL-C, low density lipoprotein cholesterol.

^*c*^Missing data: 2 for education;33 for Adl; 8 for hypertension; 32 for diabetes; expect for 905 participants without fasting, 891 for HDL-C; 899 for LDL-C; 894 for triglycerides.

Table 1 shows the relationship between different baseline characteristics and abdominal obesity. Different gender (*P* < 0.001), hypertension (*P* < 0.001), diabetes (*P* < 0.001), depression (*P* = 0.043), and biomarkers indicators have significant differences, including HDL-C (*P* < 0.001), LDL-C (*P* < 0.001) and TG (*P* < 0.001).

### 3.2 Association between abdominal obesity and cognitive decline

#### 3.2.1 Cognitive function trajectory modeling

Longitudinal cognitive function was assessed across five waves of standardized testing. The mean cognitive scores (mean ± SD) for wave 1–5 were 13.26 ± 2.75, 13.33 ± 2.74, 13.11 ± 2.73, 12.77 ± 3.24, and 13.03 ± 2.91, respectively. Detailed results (including mean values, standard deviations, and longitudinal trends) summarize in Supplementary Table 1.

**Figure 2:**
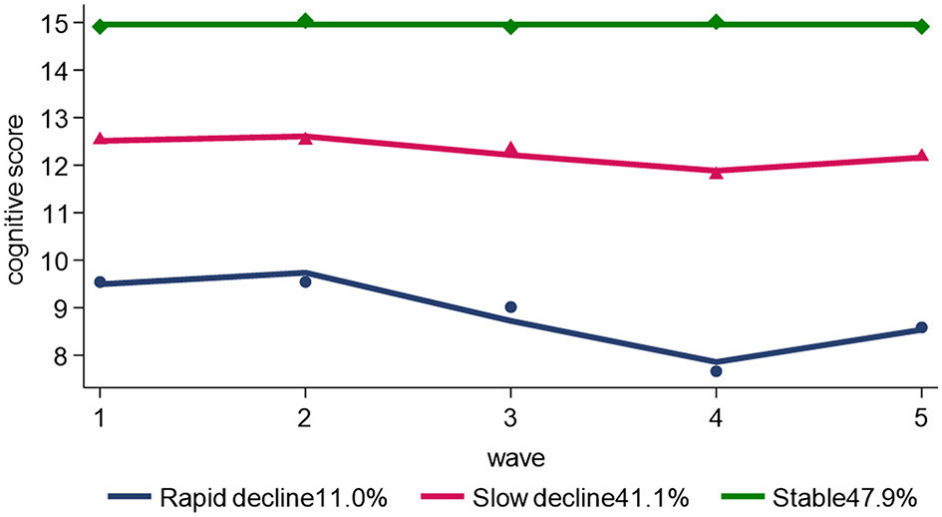
Trajectories of the cognitive scores.

By selecting the optimal *BIC* value, three distinct cognitive function trajectory models were finally fitted. The *Avepp* of the three groups were over 0.8, and the proportion of each group was >10%. The fitted trajectories (3,3,0) were rapid decline group (11.0%), slow decline group (41.1%), and stable group (47.9%) respectively. The model groupings are shown in Figure 2.

#### 3.2.2 Association between abdominal obesity and the trajectories of cognitive

Table 2 shows the relationship between abdominal obesity and cognitive function trajectory. Specifically, an increase in WC was found to be associated with a lower risk of rapid decline and slow decline (*OR*:0.98, 95%*CI*: 0.97, 0.99). Participants with abdominal obesity also showed a lower risk of rapid decline (*OR*: 0.98, 95%*CI*: 0.97, 0.99) and slow decline (*OR*:0.98, 95%*CI*: 0.97, 0.99). We observed that the impact of WC and abdominal obesity on cognitive score decline remained significant even after adjusting for potential confounding factors, such as demographic factors (age, gender, and education), lifestyle habits, health status (self-care abilities, depression and presence of chronic diseases), and biomarkers (LDL-C and TG). Taking stable group as a reference, for *1-SD* increase was associated with lower risk of rapid decline (*OR:* 0.97, 95%*CI*: 0.96, 0.98) and slow decline (*OR*:0.99, 95%*CI*: 0.98, 0.99). Compared with normal participants, those with abdominal obesity had a lower risk of rapid decline in cognitive scores (*OR*:0.67, 95%*CI*: 0.51, 0.8), and a lower risk of slow decline in cognitive scores (*OR*:0.81, 95%*CI*: 0.69, 0.95).

**Table 2:** Association between abdominal obesity with trajectories of cognitive scores.

**Variable**	**Model 1^a^**	**Model 2^a^**	**Model 3^a^**

	**OR (95%CI)** ***P***	**OR (95%CI)** ***P***	**OR (95%CI)** ***P***
**WCb Per SDb**			
RA vs. STb	0.98 (0.97, 0.99) <0.001	0.97 (0.96, 0.98) <0.001	0.97 (0.96, 0.98) <0.001
SL vs. STb	0.98 (0.97, 0.99) <0.001	0.99 (0.98, 0.99) <0.001	0.99 (0.98, 0.99) 0.001
**Normal**			
RA vs. STb	1.0	1.0	1.0
SL vs. STb	1.0	1.0	1.0
**Obesity**			
RA vs. STb	0.74 (0.60, 0.93) 0.008	0.64 (0.50, 0.81) <0.001	0.67 (0.51, 0.87) 0.002
SL vs.STb	0.81 (0.70, 0.92) 0.002	0.79 (0.68, 0.91) 0.002	0.81 (0.69, 0.95) 0.009

^*a*^Model 1 was non-adjusted; Model 2 was adjusted for age, gender, education, smoke, and drink; and Model 3 was further adjusted for adl, iadl, depression and chronic disease (hypertension, diabetes), and biomarkers (low density lipoprotein cholesterol, triglycerides).

^*b*^SD, standard deviation. WC, waist circumference; RA, rapid decline; ST, stable; SL, slow decline.

#### 3.2.3 Does–response relationship

**Figure 3:**
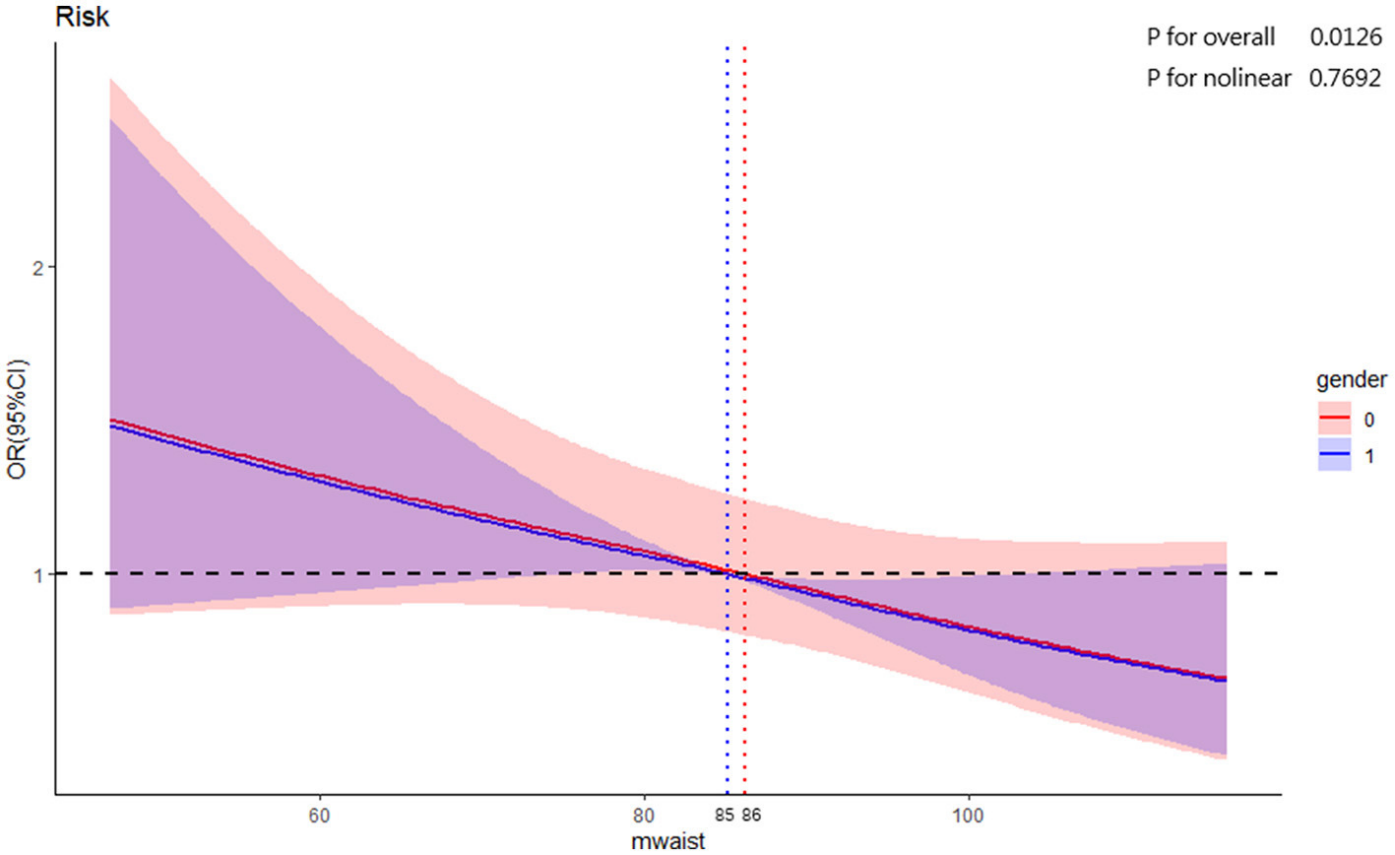
RCS for the association of baseline WC with cognitive decline.

To explore the potential nonlinear dependency between WC and the risk of cognitive decline, RCS model stratified by gender was used to create dose-response curves, with WC considered as a continuous variable. According to the Bare Pool Information Criterion, the 3-node model showed the smallest Akaike information criterion (AIC) value (4,770.65); hence, the number of model nodes was chosen to be 3, as shown in Figure 3. After adjusting for confounders, the relationship between WC and risk of cognitive decline exhibited a statistically linear correlation (*P*_overall_ = 0.0126, *P*_non − linearity_ > 0.05). The results showed that *OR* linear descending as WC increasing, and as WC approached the cut off value of 85 cm for male and 86 cm for female, *OR* was 1. Subsequently, there was a significant protective effect on cognitive function when WC exceeds the cut off value.

#### 3.2.4 Age-based subgroup analysis

Subgroup analyses revealed a significant association between abdominal obesity and cognitive decline in older adults aged 50 and above. Compared to those with normal WC, participants over the age of 50 with abdominal obesity were associated with a decreased risk of being assigned in rapid decline group by 0.38 times (*OR*:0.62, 95%*CI*: 0.43, 0.90) and slow decline group by 0.23 times (OR:0.77, 95%*CI*: 0.61, 0.97) respectively. In addition, *1-SD* increase in WC, the risk of participants assigned to rapid decline group was decreased by 0.03 times (*OR*:0.97, 95%*CI*: 0.96, 0.99) and slow decline group decreased by 0.01 (*OR*:0.99, 95%*CI*: 0.98, 1.00) respectively. However, no significant association was recognized among older adults under the age of 50. Detailed findings are presented in Table 3.

**Table 3:** Subgroup analysis between abdominal obesity and cognitive trajectories by gender.

**Variable**		**Model 1** ^**a**^		**Model 2** ^**a**^		**Model 3** ^**a**^	

		**OR (95%CI)**	***P*** **-value**	**OR (95%CI)**	***P*** **-value**	**OR (95%CI)**	***P*** **-value**
**Age**<**50**							
WC (Per SD^b^)	RA vs. ST	1.00 (0.97, 1.04)	0.816	1.00 (0.96, 1.04)	0.891	1.01 (0.96, 1.05)	0.720
	SL vs. ST	0.99 (0.97, 1.00)	0.131	0.99 (0.97, 1.00)	0.157	0.99 (0.97, 1.01)	0.261
Normal	RA vs. ST	Ref.	–	Ref.	–	Ref.	–
	SL vs. ST	Ref.	–	Ref.	–	Ref.	–
Obesity	RA vs. ST	1.70 (0.76, 3.79)	0.196	1.67 (0.68, 4.02)	0.249	1.87 (0.74, 4.73)	0.184
	SL vs. ST	0.96 (0.69, 1.34)	0.831	1.01 (0.69, 1.45)	0.969	1.07 (0.72, 1.58)	0.737
**Age** ≥**50**							
WC (Per SD^b^)	RA vs. ST	0.98 (0.97,0.99)	0.001	0.97 (0.96,0.99)	<0.001	0.97 (0.96, 0.99)	0.001
	SL vs. ST	0.99 (0.98,1.00)	0.025	0.98 (0.97,0.99)	0.013	0.99 (0.98, 1.00)	0.037
Normal	RA vs. ST	Ref.	–	Ref.	–	Ref.	–
	SL vs. ST	Ref.	–	Ref.	–	Ref.	–
Obesity	RA vs. ST	0.74 (0.56, 0.98)	0.042	0.58 (0.41, 0.81)	0.001	0.62 (0.43, 0.90)	0.010
	SL vs. ST	0.80 (0.67, 0.97)	0.023	0.75 (0.60, 0.92)	0.006	0.77 (0.61, 0.97)	0.024

^*a*^Model 1 was rude; Model 2 was adjusted for age, gender, education, smoke, and drink; and Model 3 was further adjusted for adl, iadl, depression, chronic disease (hypertension, diabetes), and biomarkers (low density lipoprotein cholesterol, triglycerids).

^*b*^SD, standard deviation. WC, waist circumference; RA, rapid decline; ST, stable; SL, slow decline.

### 3.3 Mediation analysis

**Figure 4:**
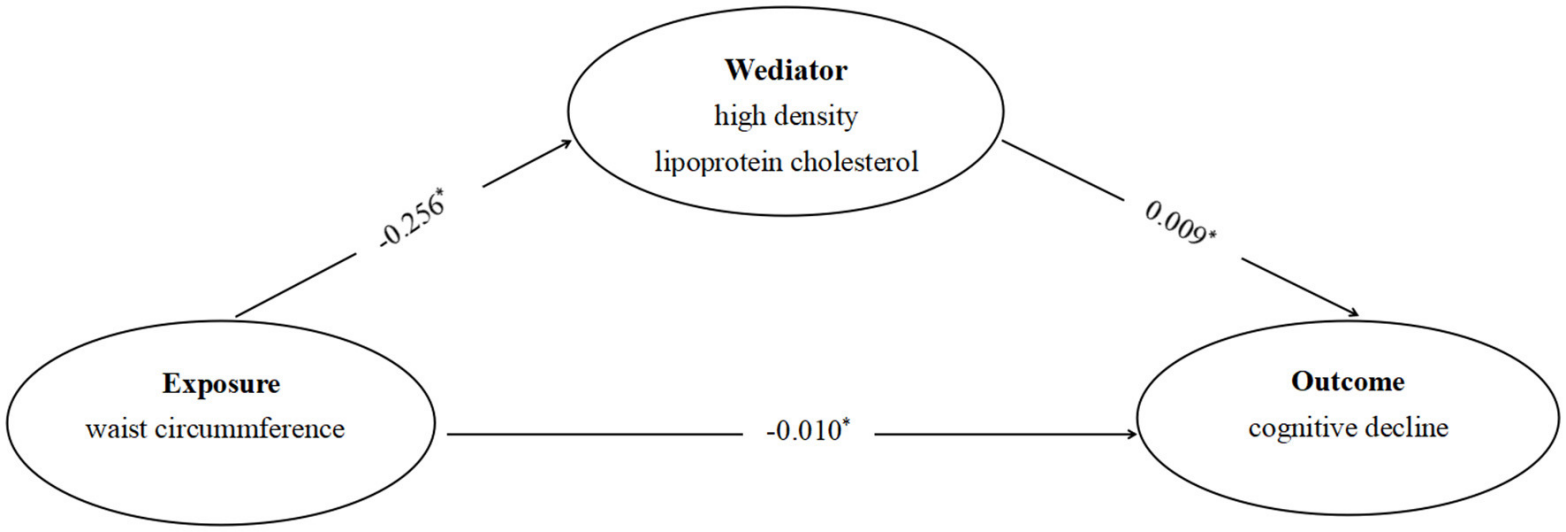
The diagram of the mediation analysis of WC on cognitive decline. ^*^P < 0.05.

This study used HDL-C as mediating variables and WC as the independent variable. We divided the 3 cognitive function trajectories into binary outcome (stable and decline groups). Covariates were sequentially adjusted to establish the model, and the Bootstrap method was employed to test the mediating effect of HDL-C between WC and cognitive function in middle-aged and older adults. The results indicated that HDL-C played a partial mediating role in the relationship between WC and cognitive function in middle-aged and older adults, with a mediation effect values for HDL-C on cognitive decline of 19.15%. Specifically, as shown in the mediation model (Figure 4), it was found that the WC had an inverse relationship with cognitive decline (β = −0.010). In addition, there was an inverse relationship between WC and HDL-C (β = −0.256), and HDL-C (β = 0.009) was an independent mediator of the detrimental effect of WC on cognitive function (Table 4).

**Table 4:** Proportion of association of WC with trajectories of cognitive mediated by HDL-C.

**Effect**	**Model 1** ^**a**^		**Model 2** ^**a**^		**Model 3** ^**a**^	

	**Estimate**	***P***	**Estimate**	***P***	**Estimate**	***P***
Total effect (TE)	−0.0022	0.002	−0.0022	0.002	−0.0021	0.006
Natural direct effect (NDE)	−0.0013	0.076	−0.0015	0.030	−0.0017	0.026
Natural indirect effect (NIE)	−0.0009	<0.001	−0.0007	0.002	−0.0004	0.016
Percentage mediated (PM)	0.4033	0.0020	0.3018	0.004	0.1915	0.022

^*a*^Model 1 was rude; Model 2 was adjusted for age, gender, education, smoke, and drink; and Model 3 was further adjusted for adl, iadl, depression, chronic disease (hypertension, diabetes), and biomarkers (low density lipoprotein cholesterol, triglycerids).

## 4 Discussion

This study conducted 5 waves of follow-up on the cognitive function trajectories of 3,807 middle-aged and older adults in China from 2011 to 2020, investigating the relationship between abdominal obesity and cognitive decline, as well as the mediating role of HDL-C. The results revealed a declining trend in cognitive decline trajectories among middle-aged and older adults in China with 3 distinct cognitive function trajectories. After gradually adjusting for confounding factors such as demographic factors, health status, and biomarkers, abdominal obesity was identified as an independent protective factor against cognitive decline. When stratified by age, abdominal obesity remained a protective factor for cognitive decline in individuals aged 50 and above. The dose-response curve revealed that WC had a linear relationship with the risk of cognitive decline, with the *OR*s decreasing as WC increased. In addition, HDL-C significantly mediated the association between WC and cognitive decline.

### 4.1 Cognitive function trajectories

Group-based trajectory model demonstrated that there is an overall decline in cognitive function among middle-aged and older adults in China, with 3 distinct cognitive function trajectories identified: stable group, rapid decline group, and slow decline group. Zhang based on CHARLS (2011–2015), including 5,693 middle-aged and older adult aged 45 and above, to conduct group-based trajectory modeling and identified the same three trajectories (27). Wang used a latent growth mixture model to estimate cognitive function changes among middle-aged and older adult, and also identified similar cognitive trajectory groups (low-level deterioration group, normal aging group, and high-level improvement group, corresponding to the rapid decline group, slow decline group, and stable group in this study) (28). Furthermore, longitudinal studies showed that the cognitive function in middle and olde-aged people decreased gradually over time, with unfavorable impairment status (29). The majority of participants had the stable trajectory (47.9%), and rapid decline group was rarest (11.0%) among the respondents. This finding was consistent with previous studies (30, 31). Therefore, to prevent and improve cognitive impairment among middle-aged and older adults, we should focus on individuals experiencing rapid declines in cognitive function for health interventions to avoid progression to dementia in order to alleviate the burden on family caregivers as well as on society's economy.

### 4.2 Association between abdominal obesity and cognitive decline

After adjusting for multiple confounding factors, regression analysis showed that the risk of rapid and slow cognitive decline in those with abdominal obesity respectively decreased by 33% (*OR*: 0.67, 95%*CI*: 0.51, 0.80) and 19% (*OR*: 0.81, 95%*CI*: 0.69, 0.95), compared to individuals with normal weight. Abdominal obesity was identified as an independent protective factor against cognitive decline. Another study from the CHARLS cohort, involving 3,035 participants aged 60 and above, also illustrated that abdominal obesity defined by WC was significantly related to a lower risk of cognitive decline (13). A meta-analysis reported that increased WC were at a reduced risk of developing Alzheimer's disease (*HR*: 0.96, 95%*HR*: 0.93, 0.99) (32). In addition, a prospective cohort study in Korea demonstrated that WC had a U-shaped correlation with cognitive function among older adults, and increased central obesity over time as assessed using WC was linked with better cognitive function in older men (33). The above research is consistent with our findings, showing that waist-defined abdominal obesity had a significant protective effect for cognitive decline among Chinese middle and older aged adults. However, the association between abdominal obesity and cognition decline has been a complex and controversial issue. Therefore, previous studies have also produced contradictory results. An longitudinal study with an 8-year follow-up period revealed that abdominal obesity in late-life carried an increase risk of cognitive impairment, compared with the low WC group, the middle and high WC groups were 80 and 90% higher incidence rates of cognitive impairment, respectively (34). The differences in findings may be attributed to the choice of study samples, duration of follow-up, and the number of potential confounding factors adjusted (35). Hence, additional further multicenter prospective cohort studies with high quality are required to clarify the association between abdominal obesity and cognitive changes.

Subgroup analysis by age revealed that the protective effect of abdominal obesity on cognitive decline was only significant in middle and old-aged adults aged 50 and above. Similar findings have been reported in previous studies. Xu et al. studied the interaction between WC and age on cognitive decline and found that after age stratification, the protective effect of abdominal obesity on cognition was only significant in older adults aged 70–94 (36). Tang et al. showed the association remained consistent among adults aged 65 and above, however there were no substantial association between high WC and cognitive decline in those under 65 (37). These results suggest that abdominal obesity as a factor influencing cognitive decline may have a stratified effect (38), possibly related to the age-dependent association between obesity and cognitive decline (39). Additionally, RCS model results indicated that when men's WC approached 85 cm and women's approached 86 cm, the risk of cognitive decline was 1. This cut-off value aligns well with the abdominal obesity threshold (85 cm for men and 90 cm for women) in the “Criteria of weight for Chinese adults” (21). Based on the association between abdominal obesity and cognitive decline, WC could acted as a low-cost, simple, and time-saving screening tool for the early identification of cognitive decline in older adults with low WC.

### 4.3 Biological mechanisms of abdominal obesity beneficial to cognition

Previous studies have confirmed the following biological pathways can explain the protective effect of abdominal obesity on cognitive decline in middle-aged and older adults: (1) Leptin secreted by adipose tissue helps regulate the hippocampal synaptic plasticity, delaying cognitive decline (40, 41). (2) Individuals with low WC may be malnourished, lacking vitamin B and brain-derived neurotrophic factor, which deteriorate the development of neurodegenerative diseases such as dementia (13, 42). (3) Older adult with abdominal obesity have higher levels of sex hormones (testosterone in males and estrogen in females), which are beneficial to improving cognition (43). This study focused to explore the mediating effect of HDL-C on the association between abdominal obesity and cognitive decline. To the best of our knowledge, this is the first study to evaluate the mediating effect of HDL-C on the above association in middle-aged and older adults. The results indicated that HDL-C mediated the association between WC and cognitive decline, explaining ~19.15% of the total effect.

Previous studies have shown that HDL-C is associated with an increased risk of cognitive decline (15, 44). Sultana et al. found a significant association of HDL-C higher than 80 mg/dl with increased cognitive decline among participants in the Aspirin in Reducing Events in the older adults (ASPREE) trial (15). A large-scale population cohort study from Denmark also confirmed that higher HDL-C levels were associated with increased risk of all-cause dementia and Alzheimer's disease (44). With various physiological functions, HDL-C are complex particles and plays an significant role in restoring synapic connections in neurodegenerative diseases such as cognitive impairment. However, at very high plasma levels, the structural components and the actions of HDL-C change, and they may hinder normal physiological functions and be detrimental to cognitive function (11). Previous studies have found a significant negative correlation between WC and HDL-C levels, indicating that individuals with larger WC tend to have lower HDL-C levels (45, 46), which greatly reduced the risk of cognitive impairment due to high HDL-C levels, thereby protecting cognitive function in middle aged and older adults. The protective effect of abdominal obesity on cognitive decline may be indirectly regulated by HDL-C as a mediator. Diet and exercise have been identified as significant regulators of HDL-C levels (47, 48). Consequently, when investigating the association between abdominal obesity and cognitive decline in Chinese middle-aged and older adults, the role of diet and exercise in mediating mechanisms should not be underestimated. In our initial attempt to incorporate exercise (physical activity) into the analysis, we faced a considerable challenge due to a large volume of missing data (~2,000 cases), which impacted the sample's representativeness. We further discussed the impact of diet and exercise on the mediating mechanisms of HDL-C in next study.

This provides a new insights into the pathogenesis of cognitive diseases such as Alzheimer's disease. Therefore, it is recommended to strengthen lipid testing for middle-aged and older adults, especially continuous monitoring of high-density lipoprotein levels for early intervention to prevent cognitive decline.

### 4.4 Strength and limitations

The current study possesses the following strengths. Firstly, it is based on the latest nationally representative database of CHARLS, tracking five waves of longitudinal data on the relationship between abdominal obesity and cognitive decline, with more repeated cognitive measurements and longer follow-up cohort compared to prior research, making the results more credible (13, 27, 35). Secondly, considering the prevalence of abdominal obesity in the middle-aged and older adults in China (5) and that body fat tends to accumulate in the abdomen with age, we used a more agile WC indicator to define abdominal obesity and analyzed its association with cognitive decline, deepening the epidemiological research on the association. Finally, this study innovatively evaluated the mediating effect of HDL-C and confirmed the underlying mechanism of HDL-C between abdominal obesity and cognitive decline. From a feasibility perspective, these findings provide multidimensional targeted intervention strategies for middle-aged and older adults and thereby presenting avenues for preventing and delaying cognitive impairment in the older adults.

Nevertheless, the study also has some limitations. Firstly, limited by baseline data, despite we adjusted the date for traditional confounding factors, such as demographic characteristics, health status and biomarkers, it is possible that residual factors such as medication usage, diet, Sirtuin 1 and the APOE4 genotype that were not captured might mystify the association between abdominal obesity and cognition. CHARLS did not collect information on the diet and genetics of participants. In future study, we may consider incorporating a broader range of potential confounding variables. Secondly, the study only included the middle-aged and older adults in China, so whether the research conclusions exhibit heterogeneity in different populations requires further examination through more diverse population studies. Thirdly, as an observational study, we cannot establish a causal relationship. Further considerations may involve utilizing Mendelian randomization or genomics methods to further elucidate the above relationship.

## Conclusion

In conclusion, our study revealed that abdominal obesity had a significant protective effect on cognitive decline in middle-aged and older adults in China. Additionally, HDL-C acted as a mediator in the relationship between abdominal obesity and cognitive decline. The results of this study had significant implications for the prevention of cognitive impairment. However, further prospective high-quality multicenter cohort studies are needed to validate the association of abdominal obesity and cognitive decline and to elucidate the underlying pathophysiological mechanisms of HDL-C. From a clinical perspective, monitoring WC status for early detection and providing blood lipid test advice may contribute to dementia prevention and promote healthy aging.

## Data Availability

The datasets presented in this study can be found in online repositories. The names of the repository/repositories and accession number (s) can be found below: https://opendata.pku.edu.cn.
