# Supplementary material for: Association between abdominal obesity and cognitive decline among Chinese middle-aged and older adults: a 10-year follow-up from CHARLS
Source: Front Public Health. 2025 Apr 15;13:1479355. doi: 10.3389/fpubh.2025.1479355 (PMC12037387; doi:10.3389/fpubh.2025.1479355)
Supplement: Supplementary file 1 [file Table_1.docx]

**Supplementary table 1** Basic situation of five waves cognitive function scores

| Wave | Year | N | | Mean | Standard deviation | Range |
| --- | --- | --- | --- | --- | --- | --- |
| 1 | 2011 | 3807 | 13.26 | | 2.75 | 2.50, 21.00 |
| 2 | 2013 | 3807 | 13.33 | | 2.74 | 3.00, 21.00 |
| 3 | 2015 | 3807 | 13.11 | | 2.73 | 1.50, 20.50 |
| 4 | 2018 | 3807 | 12.77 | | 3.24 | 1.00, 20.50 |
| 5 | 2020 | 3807 | 13.03 | | 2.91 | 1.00, 20.50 |
